# Synthesis, Optimization, and Characterization of Cellulase Enzyme Obtained from Thermotolerant *Bacillus subtilis* F3: An Insight into Cotton Fabric Polishing Activity

**DOI:** 10.4014/jmb.2309.09023

**Published:** 2023-10-18

**Authors:** Amr Fouda, Khalid S. Alshallash, Hossam M. Atta, Mamdouh S. El Gamal, Mohamed M. Bakry, Abdullah S. Alawam, Salem S. Salem

**Affiliations:** 1 Botany and Microbiology Department, Faculty of Science, Al-Azhar University, Nasr City, Cairo 11884, Egypt; 2 Department of Biology, College of Science, Imam Mohammad Ibn Saud Islamic University, Riyadh 11623, Saudi Arabia

**Keywords:** Cellulase, *Bacillus subtilis*, thermotolerant, Box-Behnken design, purification, bio-polishing

## Abstract

The efficacy of 40 bacterial isolates obtained from hot spring water samples to produce cellulase enzymes was investigated. As a result, the strain *Bacillus subtilis* F3, which was identified using traditional and molecular methods, was selected as the most potent for cellulase production. Optimization was carried out using one-factor-at-a-time (OFAT) and BOX-Behnken Design to detect the best conditions for the highest cellulase activity. This was accomplished after an incubation period of 24 h at 45°C and pH 8, with an inoculum size of 1% (v/v), 5 g/l of peptone as nitrogen source, and 7.5 g/l of CMC. Moreover, the best concentration of ammonium sulfate for cellulase enzyme precipitation was 60% followed by purification using a dialysis bag and Sephadex G-100 column chromatography to collect the purified enzyme. The purified cellulase enzyme was characterized by 5.39-fold enrichment, with a specific activity of 54.20 U/mg and a molecular weight of 439 kDa. There were 15 amino acids involved in the purified cellulase, with high concentrations of 160 and 100 mg/l for glycine and proline respectively. The highest stability and activity of the purified cellulase was attained at pH 7 and 50°C in the presence of 150 ppm of CaCl_2_, NaCl, and ZnO metal ions. Finally, the biopolishing activity of the cellulase enzyme, as indicated by weight loss percentages of the cotton fabric, was dependent on concentration and treatment time. Overall, the thermotolerant *B. subtilis* F3 strain has the potential to provide highly stable and highly active cellulase enzyme for use in biopolishing of cotton fabrics.

## Introduction

Enzymes are biological molecules defined as specialized proteins that act as biocatalysts for accelerating the chemical reaction rates within living organisms. These biocatalysts reduce the amount of energy needed for a reaction to take place, thereby enabling essential cellular processes to function more effectively [1]. Enzymes have highly precise active sites that may accommodate substrates, which in turn promotes interactions and facilitates the conversion of substrates into products [2]. Due to this specialization, enzymes are able to function with pinpoint accuracy, limiting wasteful byproducts and maximizing energy efficiency. Enzymes are useful in a wide variety of fields. For instance, proteases are used to make cheese, whereas amylases and lipases are used in the baking and flavoring industries. The pharmaceutical industry makes extensive use of enzymes as they play crucial roles in drug manufacturing and formulation. Other industries ranging from textiles and dying to paper, agriculture, bioethanol, beverages, detergent, juice production, feed, leather, and waste treatment also utilize various enzymes [2, 3].

Microorganisms, especially bacterial species, are considered the main source of enzymes because of their unique properties, which include adaptability to various environmental conditions and rapid growth. Due to these reasons, *Bacillus* species in particular are dependable workhorses in biotechnology and are highly desirable for commercial enzyme production. Their well-known capacity to synthesize and secrete a wide variety of enzymes, as well as their amenability to genetic manipulation and high yield, make *Bacillus* species efficient for low-cost, large-scale production [4, 5]. In addition, enzymes produced by *Bacillus* spp. are versatile and can function in diverse environments, including those with extreme pH and high temperature [4]. Such extremophile microorganisms have attracted attention due to the remarkable stability and functionality of their enzymes under harsh environments. The capacity of thermophilic enzymes to perform at high temperatures makes them highly valued in a wide range of biotechnological and industrial applications. These enzymes originate from bacteria that can survive in hot springs and hydrothermal vents, where temperatures can reach hundreds of degrees [6]. This is why thermophilic enzymes are so useful in industries where cleanliness is of the utmost importance, such as the food and pharmaceutical industries. When thermophilic enzymes are used, reactions can take place at higher temperatures, reducing the need for further cooling or heating, which in turn reduces energy usage [7]. Overall, thermophilic enzymes are invaluable tools in a broad variety of applications, including but not limited to biofuel production, DNA amplification techniques like PCR, and other biotechnological processes due to their flexibility and endurance to extreme temperatures [8].

Plant cell walls are composed of cellulose, a complex carbohydrate that is hydrolyzed by cellulase enzymes [9]. Cellulases formed using thermotolerant bacteria have great potential, and there are several benefits to using thermophilic bacteria like *Bacillus* spp. for cellulase synthesis. These bacteria, when grown in a lab, may secrete cellulases that are both highly effective and stable even when exposed to high temperatures [10]. Cellulase enzymes from thermotolerant bacteria are essential in the production of bioethanol and other biofuels, as they convert cellulose into fermentable sugars during the biomass conversion process. The enzymes' thermotolerance is particularly useful in these procedures since it accelerates and improves cellulose degradation, leading to greater biofuel yields [11]. Thermostable cellulases have uses in agricultural sectors and the feed industry. They improve the efficiency of feeding cattle by making more of the nutrients in cellulose-rich feed ingredients available to the animals [12]. Also, thermotolerant cellulase enzymes can be useful in bioremediation since they speed up the decomposition of organic waste and make composting easier [13]. Thermotolerant cellulases also aid in the refining and polishing of pulp in the paper industry, which reduces use of chemicals and saves energy.

Biopolishing, or the utilization of enzymes to improve and enhance the appearance and quality of cotton fabrics, is an important and eco-friendly process in textile finishing. Cellulase enzymes, specifically thermotolerants, play a crucial role in biopolishing by eliminating loosely bound and protruding cellulose fibers from the fabric surface. This process leads to smoother and softer fabrics with enhanced drapes, reduced pilling, and improved color brilliance [14]. Cellulase biopolishing is an alternative method that offers several advantages over chemical or mechanical treatments, since the former uses harmful substances and produces waste, while the latter inflicts damage and wear on the fabric, as it only affects the surface fibers. Biopolishing uses cellulases which are biodegradable and effective under less harsh conditions, and therefore they help to reduce the amount of resources needed to complete the textile finishing process, including water, energy, and chemicals [15, 16].

In the current study, we sought to investigate the activity of thermotolerant bacterial species isolated from hot springs to produce cellulase enzymes. Therefore, different samples (water and soils) were collected from hot springs and used as a source for bacterial isolation. The most potent cellulase-producer bacterial strain was selected and underwent traditional and molecular identification. The highest production conditions were assessed by the one-factor-at-a-time (OFAT) method and Box-Behnken design. The production, purification, and characterization of cellulase enzymes were investigated. Finally, the activity of cellulase in the biopolishing of cotton fabrics was studied.

## Materials and Methods

### Materials

Ammonium sulfate ((NH_4_)_2_SO_4_), Rochelle salt (sodium potassium tartrate, C_4_H_4_O_6_KNa·4H_2_O), sodium hydroxide (NaOH), sodium bisulfite, 3,5-dinitro salicylic acid, carboxymethyl cellulose (CMC) powder, 95%ethanol, Lougle’s iodine solution, o-phosphoric acid (H_3_PO_4_), ready-prepared nutrient agar, dialysis bag, Sephadex G-100, and Coomassie Brilliant Blue (CBB) were acquired from Sigma Aldrich (Egypt). All reactions were achieved using sterilized distilled water (dH_2_O).

### Sampling and Bacterial Isolation

Soil and water samples, essential for bacterial isolation, were collected from Ain-Helwan spring (Egypt) (N 29°51', E 31° 19') in sterilized falcon tubes. The temperatures and pH values of the water samples were recorded upon collection, ranging from (40° to 45°C) and (6.7 to 7.14), respectively. Subsequently, the water and soil samples were quickly transported to the laboratory for bacterial isolation.

To begin the isolation process, one gram of each soil sample was dispersed in 10 ml of sterile dH_2_O. Additionally, 1 ml of the soil suspension or collected water samples was transferred to 9 ml of dH_2_O, and this procedure was repeated to perform serial dilution, ranging from 10^–1^ to 10^–5^. From the 10^–5^ dilution, 100 μl was spread onto the surface of nutrient agar plates (containing g/l: beef extract 3.0, peptone 5.0, NaCl 0.5, agar 20.0, and dH_2_O1 L; pH 7), which were then incubated at 45°C for 24 h. Following incubation, the bacterial colonies that emerged on the plates were carefully collected using a sterilized needle and re-inoculated onto new nutrient agar plates to ensure purity. The purified bacterial isolates were further cultured on nutrient agar slants and preserved at –4°C for subsequent study.

### Screening of Cellulase-Producing Bacteria

The purified bacterial isolates were investigated for their potential to produce cellulase enzyme on mineral salt agar (MSA) media (containing g/l: KH_2_PO_4_, 1; NaNO_3_, 5; MgSO_4_.7H_2_O, 0.5; K_2_HPO_4_, 2; CaCl_2_, 0.01; KCl, 0.1; FeSO_4_.7H_2_O, 0.02; agar, 20, dH_2_O, 1 L; pH 7) supplemented with 5 g (w/v) CMC powder [17]. The MSA media was poured into sterilized Petri dishes, where a pure bacterial isolate was inoculated in the center of each plate before incubation for 24 h at 45°C and flooding with Louglés iodine solution. The ability of a bacterial isolate to secrete cellulase enzymes is revealed in the form of a distinct zone around the bacteria's growth. The findings quantified the size of a clear zone in mm. The experiment was performed in triplicate for accuracy.

### Identification of the Most Potent Cellulase-Producing Strain

The most potent cellulase-producing bacterial strain was primarily identified using physiological, biochemical, and microscopic examination. Gram-reaction, enzymatic activity (urease, coagulase, catalase, and oxidase test), O/F (oxidation/fermentation) test, carbohydrate fermentation, methyl red test, indole test, citrate utilization, Voges-Proskauer test, and utilization of some substrates such as gelatin and starch were achieved for traditional identifications based on standard key [18].

To confirm traditional identification, the amplification and sequencing of the 16s rRNA gene region were performed using universal primers of 27f (5'-AGA GTT TGA TCC TGGCTC AG-3') and 1492r (5'-GGT TAC CTT GTT ACG ACTT-3') [19]. The PCR tube was filled with the following components: 0.5 mM MgCl_2_, 1×PCR buffer, 2.5U Taq-DNA polymerase (Qiagen, Country), 0.5 μM of universal primer, 0.25 mM dNTP, and 5 ng of genomic DNA. The analysis was performed using the following protocol: an initial denaturation step at 94°C for 3 min, followed by 30 cycles of denaturation at 94°C for 0.5 min, annealing at 55°C for 0.5 min, extension at 72°C for 1 min, and a final extension step at 72°C for 10 min [20]. The PCR result was sequenced (forward and reverse) using the 3730xl DNA Analyzer technology from Applied Biosystems (Egypt) at Sigma. NCBI's BLAST nucleotide was used to verify whether the retrieved sequence was similar to any in the GenBank database. The phylogenetic tree was built using the neighbor-joining method in MEGA (Version 6.1) software at a confidence level of 1000 replicates.

### Estimation of the Cellulase Activity and the Protein Concentration Quantitatively

The cellulase enzyme activity was estimated by measuring the amount of reducing sugars that were produced due to the breakdown of the CMC by using the dinitro salicylic acid method (DNS). The DNS reagent was prepared as follows: dinitrodicylic acid, 6.3 g/l; Rochelle salts, 182 g/l; phenol, 5.7 g/l; sodium bisulfite, 5 g/l; and NaOH, 21.4 g/l [21]. The red color formed once the DNS bound to reducing sugars. During the assay, 1 mL of 1%(w/v) CMC (prepared in phosphate buffer, pH 7) was added to a test tube and mixed well with 200 μl of culture filtrate. The mixture was incubated at 45°C for 30 min before being mixed with 1 ml DNS reagent. The previous mixture was introduced to a water bath at 100°C for 5 min. After cooling, the color intensity was measured by using spectrophotometry at a wavelength of 540 nm. By using a standard curve (prepared from various concentrations of glucose), the amount of reducing sugars released was measured. One unit of cellulase enzyme activity was defined as the amount of enzyme that released 1 μmol/min of reducing sugar. The total soluble protein content was measured by the Bradford method [22] using Coomassie Brilliant Blue (CBB) G-250. The CBB was prepared by dissolving the CBB dye in 50 mL of 95% ethanol. A total of 100mL of o-phosphoric acid was added later and the whole reagent was diluted to 200 mL to make 5 × concentrated dye. In this method, the soluble protein was reacted with the CBB reagent leading to a change in the reaction color from a pale green to a blue color. The formed color was measured through using spectrophotometry at a wavelength of 595 nm.

### Optimization of Cellulase-Producing Conditions

**OFAT Method**

The bacterial strain *B. subtilis* F3 was subjected to different environmental incubation conditions to identify the optimal parameters that would yield the highest cellulase production. First, the optimization process was achieved using the one-factor-at-a-time (OFAT) method. To achieve this objective, a mineral broth medium was prepared and enriched with CMC powder, followed by autoclaving and inoculation with 2% (v/v) of an overnight bacterial culture after cooling. Throughout the experiment, multiple environmental factors were explored, according to the OFAT method, such as pH levels (6 to 10), temperatures (30°C to 50°C with regular intervals of 5°C), inoculum sizes (1% to 4%, v/v), incubation periods (6 to 30 h with regular intervals of 6h), various nitrogen sources (ammonium nitrate (NH_4_NO_3_), ammonium chloride (NH_4_Cl), ammonium hydrogen orthophosphate ((NH_4_)_3_PO_4_), beef extract, urea, and peptone), and different CMC concentrations (2.5 g/l to 10 g/l with regular interval concentration of 2.5 g/l).

After each factor's incubation period had ended, about 2 ml of the inoculated medium was withdrawn and centrifuged (4°C for 10 min at 10,000 ×*g*). The resulting supernatant was then collected and utilized to detect cellulase activity through the DNS method. Each factor was achieved by keeping all other parameters under optimum conditions. The experiment for each factor was carried out in triplicates.

### Box-Behnken Design

To attain the maximum productivity of the cellulase enzymes, Box-Behnken design (BBD) was used accordingly for data obtained from OFAT. In this method, six variable parameters including temperature, inoculum sizes, pH, incubation period, CMC concentrations, and nitrogen sources were investigated within three levels (+1, 0, -1) and shown in Table 1. BBD was employed to generate higher-order response surfaces more efficiently than traditional factorial methods, requiring fewer experimental runs. This design, known for its broad applicability, was specifically utilized to create quadratic response surfaces and establish a second-degree polynomial model for analyzing enzyme production patterns. The optimization process was subsequently carried out through a series of experimental runs based on this polynomial model [23].

To ensure the accuracy of the higher-order surface representation, BBD omits specific runs in its implementation (version 12, Stat-Ease Inc., USA). This statistical approach is applied to optimize cellulase activity, and the number of experimental runs (N) is determined based on the equation N = k2 + k + Cp, where k represents the number of factors, and Cp denotes the number of replications. In the present study, a second-degree polynomial equation was utilized, resulting in a predicted model derived from 54 distinct experimental runs. The current work employed this second-degree polynomial equation, which also led to 54 different experimental runs in total. The second-degree polynomial equation for the predicted model can be represented as follows:

Y=X_0_ + X_1_A + X_2_B + X_3_C + X_4_D + X_5_E + X_6_F + X_12_AB + X_13_AC+ X_14_ AD + X_15_AE + X_16_ AF + X_23_BC + X_24_BD + X_25_BE+ X_26_ BF+ X_34_ CD+ X_35_ CE+ X_36_ CF+ X_45_ DE+ X_46_ DF+ X_56_ EF+ X_11_A_11_ + X_22_B_22_ + X_33_C_33_+ X_44_D_44_ + X_55_E_55_ + X_66_F_66_,

where Y is the response (cellulase production); X_0_ is the constant value; A, B, C, D, E, and F are the free factors; X_1_, X_2_, X_3_, X_4_, X_5_, and X_6_ are the direct coefficients; X_12_, X_13_, X_14_, X_15_, X_16_, X_23_, X_24_, X_25_, X_26_, X_34_, X_35_, X_36_, X_45_, X_46_, and X_56_ are the cross product coefficients, and X_11_, X_22_, X_33_, X_44_, X_55_, and X_66_ are the quadratic coefficients.

### Extraction and Purification of Cellulase Enzyme

All the extraction and purification steps of cellulase enzymes were carried out at 4°C. After cultivation, the inoculated mineral salt (MS) broth underwent centrifugation at 5,000 ×*g* for 15 min followed by mixing with ammonium sulfate ((NH_4_)_2_SO_4_) at varied concentrations (10% to 80%). This step aimed to concentrate the cellulase enzyme in the cell-free supernatant. Subsequently, the ammonium sulfate fractions were collected by centrifugation at 10,000 ×*g* for 30 min and dissolved in phosphate buffer with a pH of 7.0. The resulting turbid suspension was further centrifuged for 10 min at 10,000 ×*g*, resulting in a clear supernatant [24].

The collected crude supernatant obtained from the (NH_4_)_2_SO_4_ step was subjected to more purification using a dialysis bag by putting it in a cup against dH_2_O for 3 h, followed by transferring the dialysis bag content to phosphate buffer (pH 7). To concentrate the cellulase enzyme, the crude cellulase in a dialysis bag was put against sucrose crystal in a cup and kept in a refrigerator at 4°C for complete purification using Sephadex G-100 column chromatography.

The column (1.5 × 50 cm) was filled with Sephadex G-100 and equilibrated with phosphate buffer (pH 7) before being loaded with the cellulase enzyme obtained from a dialysis bag. The fractions with the highest cellulase activity from the ion exchange chromatography step were applied to the column. Each 5 ml from the flow rate column (5 ml/h) was collected to detect the activity of cellulase in each fraction.

### Cellulase Enzyme Characterization

**Detection of Molecular Weight by TLC Mass Spectroscopy**

In the current study, the molecular weight of purified cellulase enzyme was detected by using the Advion compact mass spectrometer (CMS, USA). Different ion sources were used, including ESI, APCI, and APCI/ASAP. The analysis allows for switching between positive and negative ion polarity in a single analysis. The flow rate range for ESI is 10 μl/min to 1 ml/min, and 10 μl/min to 2 ml/min for APCI. The m/z range for Expression S is 10 to 1,200, and 10 to 2,000 for Expression L. The sensitivity of ESI is demonstrated by achieving a 10 pg reserpine signal-to-noise ratio (S/N) of 100:1 (RMS) with SIM of m/z 609.3. Additionally, a standard substance, sulphadiazine (M. Wt = 250), was injected to ensure the quality of the analysis.

### Identification of Amino Acid Content

The amino acids of the cellulase enzyme were detected by Sykam Amino Acid Analyzer (Sykam GmbH, Germany) equipped with an Autosampler S 5200, Amino Acid Reaction Module S 4300 (with built-in dual filter photometer between 440 and 570 nm with constant signal output and signal summary option), a Refrigerated Reagent Organizer S4130 and a Solvent Delivery System S 2100 (with a quaternary pump having a flow range of 0.01 to 10.00 kl/min and maximum pressure up to 400 bar). Standard preparation was used, where the stock solution contains 18 amino acids (aspartic acid, threonine, serine, phenylalanine, histidine, lysine, arginine, glutamic acid, proline, glycine, alanine, cystine, valine, methionine, isoleucine, leucine, and tyrosine). The concentration of all amino acids was 2.5 μMol/ml (except for cystine at 1.25 μMol/ml). Next, 60 μl for dilution in a 1.5-ml vial of sample dilution buffer was subjected to a filtration process using a 0.22-μm syringe filter, and then 100 μl was injected. Sample preparation was achieved as follows: 1 g of the sample was mixed with 5 ml hexane and left to macerate for 24 h. Then, the mixture was filtered by using a Whatman No. 1 filter paper, and the residue was transferred into a test tube where it was incubated in an oven with 10 ml 6N HCl for 24 h at 110°C. After incubation, the sample was filtered on Whatman No. 1 filter paper, evaporated on a rotary evaporator, and dissolved completely in 20 ml dilution buffer. Then, the sample was filtered again using a 0.22-μm syringe filter, and 100 μl was injected.

### Effect of Temperature, pH, and Metal Ions on the Stability of Cellulase Enzyme

The effect of some metal ions, pH, and temperature on cellulase activity and stability was investigated. The assay method was carried out by incubating the purified cellulase enzyme at different pH values (5–10), different temperatures (30–80°C), and with different concentrations (50, 100, 150, and 200 ppm) of metal ions, including CaCl_2_, NaCl, ZnO, FeCl_3_, CdCl_2_, HgCl_2_, and CuSO_4_.5H_2_O. At the end of each factor, 100 μl of purified cellulase was added to 1 ml of (1%, w/v) CMC on phosphate buffer (pH 7), and incubated for 1 h. The activity of cellulase after incubation at different temperatures, pH, and metal ions, was measured by the DNS method [25].

### Application of Cellulase on Biopolishing of Cotton Fabric

The application of bacterial cellulase enzyme to cotton fabrics was carried out in a variety of concentrations (0.25, 0.5, and 1%) for various durations (30, 60, and 90 min). The weight loss of cotton fabrics treated with cellulase was detected. After each course of treatment, the amount of weight reduction was determined by applying the formula below [16]:

$$\text{Weight loss \%=}\frac{\text{A-B}}{\text{A}}\text{×100,}$$

where A is the weight of cotton fabric before treatment, and B is the weight of cotton fabric after cellulase treatment.

### Statistical Analysis

All results in the current study were the means of three independent replicates. Data were introduced by one-way analysis of variance (ANOVA) by the statistical package Minitab v19. The mean difference comparison between the treatments was analyzed by the Tukey HSD at *p* < 0.05.

## Results and Discussion

### Isolation and Identification of Bacterial Isolates

In the present study, the bacterial strain designated as F3 was selected after checking the activity of 40 bacterial isolates obtained from water and soil samples to determine the most potent bacterial strain for cellulase activity. The first survey by agar plate method showed that the activity of 40 bacterial isolates varied according to the clear zone formed around the bacterial cells after flooding the growing plates with Logule’s iodine solution. As shown, 55% of the total bacterial isolates have the potential to produce cellulase enzymes with varied clear zones. This percentage was classified as follows: 63.6% (14 isolates) have the efficacy to produce clear zones in the range of 20–30 mm; 31.8% (7 isolates) produced clear zones with ranges of 10–20 mm, and 4.5% (one isolate) produced the largest clear zone in the range of 30–40 mm (Table 2). On the other hand, 18 bacterial isolates among the total number of 40 isolates lack cellulase activity.

Therefore, the bacterial isolate named F3 was selected as the most potent and therefore used to complete the study. In a similar study, 40 bacterial isolates obtained from soil samples showed varied cellulase activity detected after flooding the cultured plates with Congo red and measuring the clear zones formed around the bacterial growth [26]. Also, among 43 bacterial isolates, 3 strains showed high cellulase activity based on clear zones formed after staining of CMC plates with Logule’s iodine solution [27]. On the other hand, 24 bacterial isolates were obtained from different soil samples and investigated for their efficacy in cellulase production. Out of 24 isolates, 6 strains (25%) showed potential for cellulase production, as confirmed by the appearance of clear zones on the CMC agar plate when flooded with iodine stain [13].

Traditional identification showed that the bacterial strain F3 is a gram-positive, bacilli-shaped strain arranged in pairs or chains. It is also motile, spore-forming, and aerobic or facultatively anaerobic. The strain has the efficacy to grow at a wide range of temperatures (30–50°C) but lacks the potential to grow at 20°C. In addition, it is capable of producing acids from fermentative glucose, L-arabinose, D-mannitol, and sucrose, while being incapable of acid production from D-xylose and lactose fermentation. The strain showed positive results for catalase, oxidase, citrate utilization, Voges-Proskauer, starch hydrolysis, and nitrate reduction, whereas the indole test, methyl red test, and urease enzyme tests revealed negative results.

**Fig. 1:**
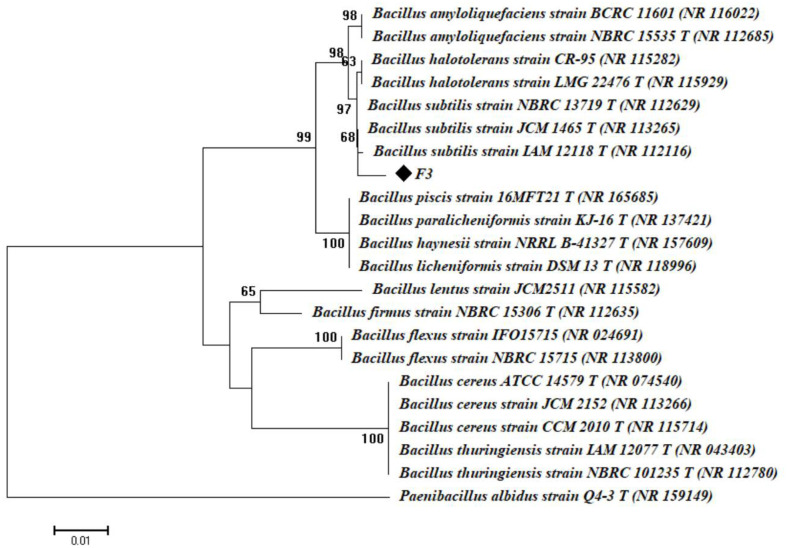
Phylogenetic tree of the bacterial strain *Bacillus subtilis* F3 based on a comparison of its 16s rRNA sequences with reference sequences from the NCBI. The study was done with MEGA 6, and the neighbor-joining method with a bootstrap value of 1000 was used.

The above results were confirmed by amplification and sequencing of the 16S rRNA gene. Based on the obtained data, the bacterial isolate F3 was similar to *B. subtilis* (closest accession number: NR113265) with a similarity of 99.39%. Therefore, the bacterial strain F3 was identified as *B. subtilis* and the sequence was deposited with GenBank (accession number ON060660). The phylogenetic analysis of *B. subtilis* strain F3 is shown in Fig. 1. Similarly, the bacterial strain *B. subtilis* CD001 was used for cellulase enzyme production using CMC as a fermentation substrate [28]. To the best of our knowledge, a few published studies have described the isolation of *B. subtilis* from hot spring samples and its use in the production of thermotolerant cellulase enzymes, and the findings increase the importance of the current study.

### OFAT Optimization

F3, the bacterial strain with the highest cellulase production, was selected and subjected to optimization of incubation conditions. Different culture conditions such as pH, temperature, inoculum size, incubation period, nitrogen source, and CMC concentration, were investigated to improve the cellulase production. Also, since the development of the media culture conditions leads to evaluation of the fermentation technology, the current study improved the fermentation conditions to realize the maximal cellulase production.

**Fig. 2:**
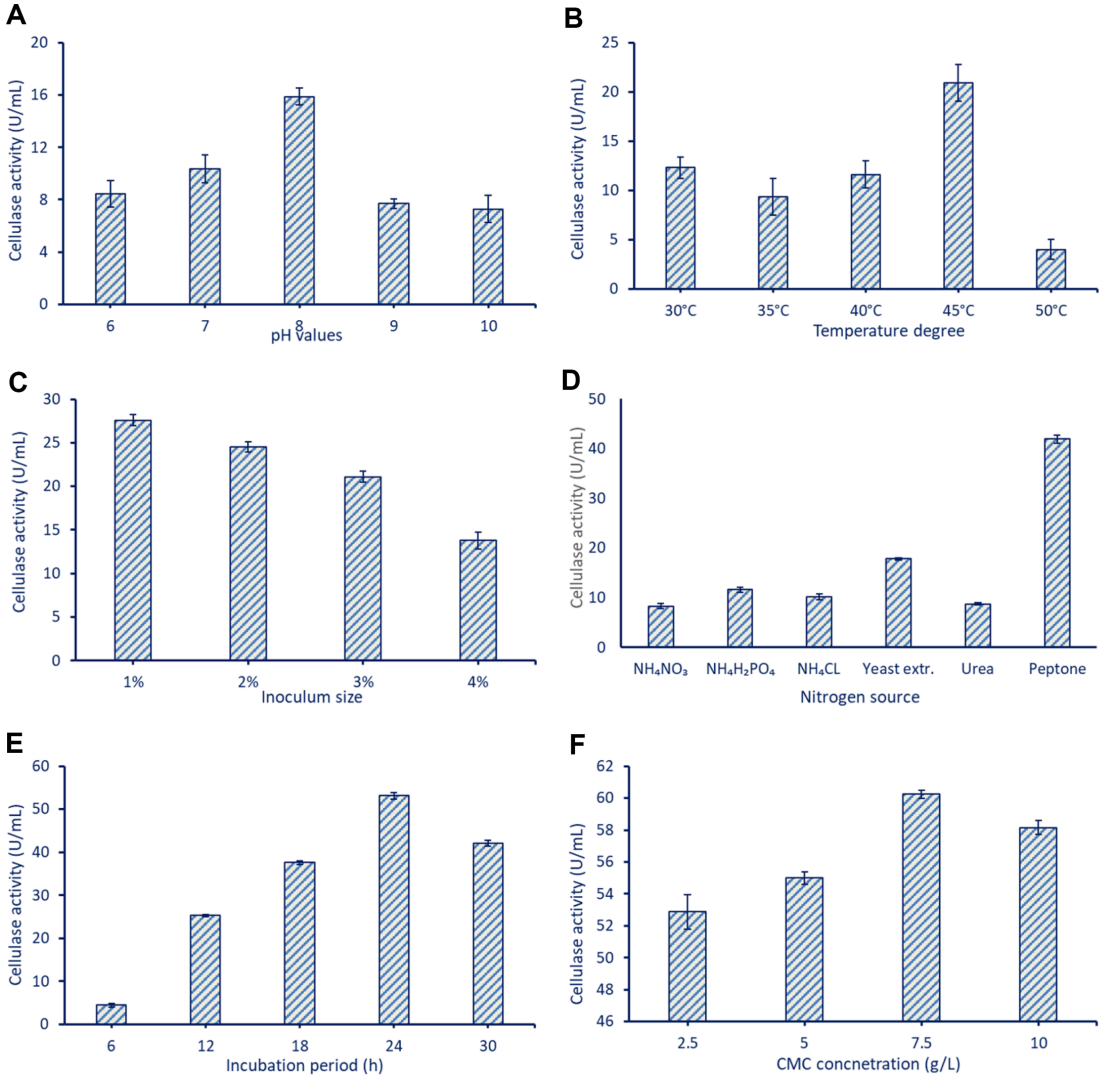
OFAT optimization of cellulase enzyme activity using *B. subtilis* F3. **A** denotes the effect of various pH values, **B** denote the different temperatures degree, **C** denote the effect of various inoculum sizes, **D** denote the effect of different nitrogen sources on the cellulase enzyme activity, **E** refer to the effect of different incubation peroid, and **F** is the effect of different CMC concentrations on the enzyme activity.

Data represented graphically in Fig. 2A showed the effect of different pH values on the productivity of cellulase enzymes. As shown, the bacterial strain *B. subtilis* F3 was able to produce the maximum amount of cellulase at a pH of 8.0, where the maximum cellulase activity was 15.9 ± 0.9 U/ml. According to the obtained data, it can be concluded that the acidic, neutral, and alkaline conditions harm F3 growth and hence cellulase production. This phenomenon could be related to the loss of the functional shape of the enzymes, which leads to altering of their ionic strength at varied pH values. At unfavorable pH values, the enzymes were subjected to denaturation due to a change of ionic state [29]. Furthermore, the enzyme's active site is highly binding with its substrate at an alkaline pH level. In a similar study, the optimum pH of culture conditions for maximum cellulose breakdown using *Bacillus* spp., and hence secretion of cellulase enzyme, was in the range of 7–9 [30]. In contrast, the maximum cellulase activity (19 U/mg protein) produced by *Bacillus* sp. was attained at pH 6 [31]. In general, the variation in optimal pH value between bacterial species during cellulase enzyme production could be due to the variation in media components and bacterial strain [32].

Temperature represents another critical factor that impacts bacterial growth and, consequently, cellulase activity. To explore the influence of various temperature levels, the bacterial strain *B. subtilis* F3 was introduced into MS broth media enriched with CMC and incubated for 24 h at different temperatures (30, 35, 40, 45, and 50°C). Data shown in Fig. 2B reveal that the maximum cellulase production was noticed at 45°C with the highest cellulase activity of 20.9 ± 1.9 U/ml. The cellulase activity is reduced with increasing or decreasing the temperature degree. This means that at a high temperature (45°C), the enzyme's kinetic energy is optimized, leading to faster enzymatic reactions. Furthermore, the enzyme's structure and activity remain intact, ensuring optimal performance [12]. In a similar study, the maximum cellulase production using *B. subtilis* strain M-11 was achieved at a temperature of 42°C [12, 33]. In contrast, the maximum cellulase production was obtained using *Bacillus* sp. at a temperature degree of 35°C, and the activity was highly decreased at a temperature above 40°C [34]. As reported previously, the optimal incubation temperature for maximizing enzyme production differs among *Bacillus* species and is influenced by various environmental and cultural factors [35].

As depicted in Fig. 2C, the maximum cellulase activity (27.6 ± 0.1 U/ml) produced by bacterial strain *B. subtilis* F3 was achieved at an inoculum size of 1% (v/v). However, as the inoculum sizes increased, the cellulase enzyme activity decreased. Specifically, the enzyme activity reached 24.6 ± 0.3 U/ml, 21.1 ± 0.1 U/ml, and 13.8 ± 0.8 U/ml at inoculum sizes of 2, 3, and 4%, respectively. Resource competition and substrate inhibition are two possible explanations for this observation. Larger bacterial inocula may result in limited space and fewer nutrients for the bacteria to grow and reproduce. As a result, cellulase activity may be diminished when the bacterial community competes for limited resources, decreasing metabolic activity and enzyme output [36]. The main function of cellulase enzymes is to hydrolyze their substrate, cellulose, into simpler sugars [37]. At higher bacterial inoculum sizes, the concentration of bacteria and their metabolic byproducts increases. This leads to a higher concentration of end products, such as cellobiose or glucose, which have the ability to inhibit cellulase activity [38]. At sufficiently high concentrations, these by-products may compete with cellulase enzymes for access to the cellulose substrate, hindering the enzymes' catalytic function. As a result, the overall cellulase activity diminishes due to substrate inhibition. Consistent with the obtained results, Abada *et al*. [11] reported that the maximum cellulase activity (19 IU/ml) produced by bacterial strain *B. albus* was attained at an inoculum size of 3% (v/v). On the other hand, cellulase enzyme was more active at an inoculum size of bacterial strain *B. licheniformis* 1-1v with a concentration of 1% [39].

To achieve effective utilization of cellulose and promote extracellular cellulase enzyme production, the fermentation media should include an external nitrogen source. Therefore, the impact of various nitrogen sources, such as NH_4_NO_3_, NH_4_H_2_PO_4_, NH_4_Cl, yeast extract, urea, and peptone, was evaluated for the effect on cellulase production after 24 h of incubation. The obtained results revealed that the highest cellulase activity (41.9± 0.1 U/ml) produced by bacterial strain F3 was achieved when peptone at a concentration of 5 g/l was present, while the incorporation of other nitrogen sources resulted in decreased enzyme activity (Fig. 2D). Peptonés nitrogen is in an organic form, making it more easily absorbed by bacteria than inorganic nitrogen sources such as NH_4_NO_3_, NH_2_H_2_PO_4_, and NH_4_Cl. The organic nitrogen in peptone can be rapidly metabolized and absorbed by microbial biomass, stimulating cell development and metabolic activity. Peptonés bioavailability and nutritional balance aid in the development and metabolism of bacteria, which in turn boosts cellulase enzyme production. Therefore, peptone-containing fermentative media have higher cellulase activity than the other nitrogen sources tried. Peptone is an organic nitrogen source rich in amino acids, proteins, peptides, inorganic salts, lipids, vitamins, and sugars. Therefore, it provides a diverse range of nitrogen-containing compounds and essential elements, making it a readily available and balanced source of nutrients for bacterial growth and enzyme production [40]. Peptonés abundance of peptides and amino acids ensures that bacteria have access to a wide variety of nitrogen sources for growth, as well as a steady supply of the building blocks for protein synthesis and enzyme manufacturing. These findings align with the results reported by Lugani and coauthors, who observed that the best nitrogen source in the fermentative media of *Bacillus* sp. Y3 for the production of cellulase enzyme is peptone [41].

In addition, to determine how long of an incubation period affects cellulase activity, the fermentative media under all previously optimized conditions were inoculated with strain *B. subtilis* F3 and left to sit at optimum temperatures for 6, 12, 18, 24, and 30 h. Fig. 2E shows that the maximum cellulase activity (53.1 ± 0.04 U/ml) was achieved at an incubation period of 24 h. There was a clear downward trend in cellulase activity with both longer and shorter incubation periods. The initial 6 h of incubation resulted in the lowest cellulase activity for this strain (4.5 ± 0.2 U/ml). This finding could be due to the bacterial strain remaining at a lag phase for a time, during which it adapts to the new environment and initiates the metabolism of available nutrients. Subsequently, an exponential phase follows, characterized by a rapid increase in the population of bacterial strains. However, after reaching the optimal incubation period, the cellulase activity starts to decrease. This decline is attributed to the decrease in metabolic activity of bacterial species, which occurs due to the reduction of nutrients or the accumulation of harmful metabolites in the growth media [42]. Therefore, it can be concluded that the accumulation of toxic metabolites or a lack of adapted nutrients in the growth media can contribute to a fall in cellulase activity.

The concentration of enzyme precursor (CMC) in the fermentative media plays a crucial role in enhancing enzyme activity. For instance, the maximum cellulase activity (60.2 ± 0.3 U/ml) using the *B. subtilis* F3 strain was achieved at a CMC concentration of 7.5 g/l (Fig. 2F). The cellulase activity gradually decreased at a concentration above and below this concentration due to the substrate inhibition phenomenon [43]. For instance, the cellulase activity was 52.9 ± 1.1 U/ml, 55.0 ± 0.4 U/ml, and 58.2 ± 0.4 U/ml at a CMC concentration of 2.5, 5, and 10 g/l (Fig. 2F). It is possible that at low CMC concentrations, there is not enough substrate available to fully support bacterial growth and hence cellulase activity. On the other hand, the substrate inhibition phenomenon occurs at large concentrations of CMC. The accumulation of final or intermediate metabolites caused by an oversupply of the CMC can block the cellulase enzymes by binding to their active sites or reducing their catalytic activity leading to less enzyme activity [44]. Thus, it follows that optimizing the ratio of substrate concentration to enzyme activity is essential. Enzyme activity may suffer if the substrate concentration is too low or too high, respectively, because of insufficient substrate or substrate inhibition. Additionally, it is worth mentioning that the ideal CMC concentrations may vary based on factors such as type of microorganism, cellulase enzyme, and experimental settings, resulting in potential changes compared to the published literature.

### Optimization Using Box-Behnken Design

**Fig. 3:**
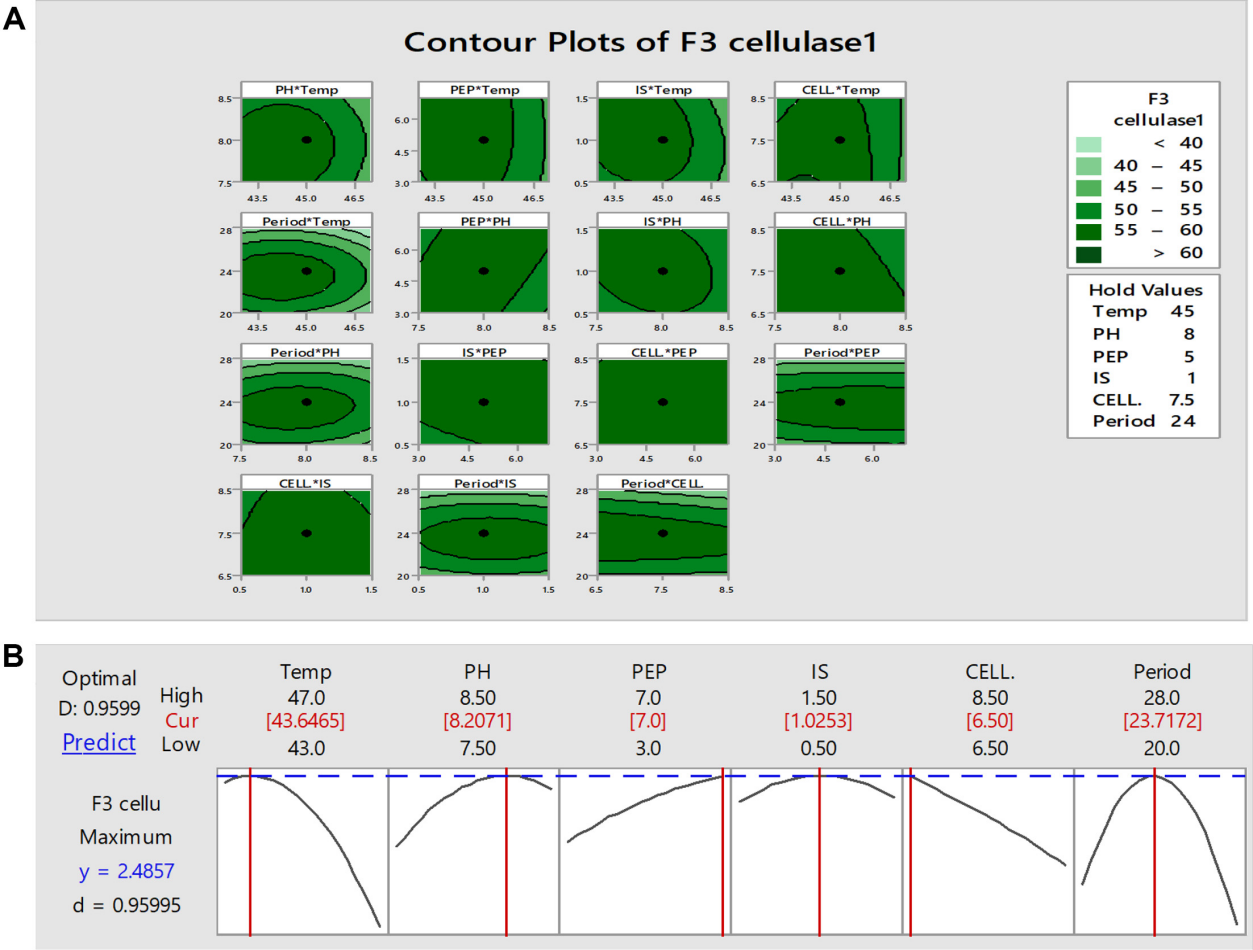
A is the interplay of various factors to determine the optimum level of each factor for maximal cellulase production using the F3 strain, and B confirms the concurrence between predicted and actual cellulase production by the bacterial strain F3. Temp is the temperature degree, pH is the pH values, PEP is the peptone as the best nitrogen source, IS refer to the inoculum size, CELL. refer to the CMC concentration, and Period denote the incubation period (h).

The optimal six factors obtained from the OFAT method, including pH, temperature, inoculum size, nitrogen source (peptone), incubation period, and CMC concentration, were subjected to Box-Behnken design (BBD) to study the effect of these factors with six levels affecting cellulase production from *B. subtilis* F3 strain. The cellulase activity ranged from 35.49 to 63.11 U/ml in all runs, as shown in Table 3 and Fig. 3A. The regression equation in uncoded units is as follows:

Y_cellulase activity_ = -3125 + 95.6 A + 178.7 B - 6.3 D + 149.2 C - 9.8 F + 29.E - 1.177 A*A - 12.38 B*B - 0.180 D*D - 8.25 C*C - 0.30 F*F - 0.6971 E*E + 0.37 A*B - 0.181 A*D - 1.537 A*C + 0.980 A*F + 0.024 A*E + 2.65 B*D- 5.98 B*C -2.77 B*F + 0.580 B*E - 1.03 D*C - 0.272 D*F - 0.061 D*E - 0.77 C*F - 0.166 C*E- 0.295 F*E,

where A = temperature, B = pH, C = inoculum size, D = nitrogen source, E = incubation period, and F = CMC concentration.

The significance of the second-order polynomial equation for cellulase activity was measured by ANOVA. From this study, the optimum factors which give the best cellulase production from the F3 strain in run 35 were a temperature of 43°C, pH (8), peptone (5 g/l), inoculum size (1.5%), CMC concentration (6.5 g/l), and incubation period (24 h). At these factors, the cellulase activity was 63.11 U/ml. Maximum cellulase activity synthesized by *B. subtilis* M-11 was measured to be 26.26 IU/gds once optimizing environmental incubation conditions using BBD at conditions of 72 h incubation, a pH of 7.5 and a temperature of 42°C [33]. On the other hand, the maximum cellulase activity (507.99 U/gds) was attained after optimizing the productivity conditions using BBD, in which the optimal conditions recorded were temperature (34°C), pH (5), moisture (60%), and incubation period (12 h.)[45]. Here, the experimental model constructed is highly significant and precisely reflects the actual relationship between the factors and the enzyme response associated with cellulase activity, as confirmed by ANOVA. The stability of the cellulase activity of the cellulolytic bacterial strain *B. subtilis* F3 is evident in the expected vs. actual plots and the normal residual plots (Fig. 3B). The distribution of response variables from various experimental conditions indicated that most of the components made similar contributions. Moreover, the likelihood graphs demonstrated a close resemblance between the expected and actual cellulase activities.

### Cellulase Enzyme Purification

**Fig. 4:**
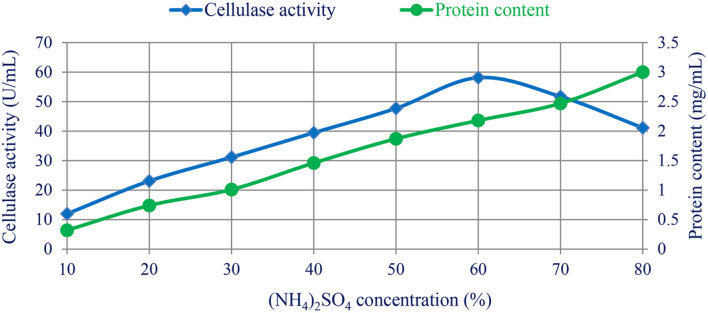
Effect of different (NH_4_)_2_SO_4_ concentrations on cellulase activity and protein content enzyme synthesized by *B. subtilis* strain F3.

The cellulase enzyme was produced using bacterial *B. subtilis* F3 strain under broth-state fermentation conditions. The protein was precipitated by different concentrations of (NH_4_)_2_SO_4_ from 10% to 80%, where the cellulase activity increased with increasing the concentration of (NH_4_)_2_SO_4_ till a concentration of 60% of (NH_4_)_2_SO_4_. The maximum cellulase activity at 60% (NH_4_)_2_SO_4_ was 58.1 ± 0.6 U/ml. The cellulase activity decreased with increasing or decreasing the optimal concentration. The total soluble protein increased with increase in the concentration of (NH_4_)_2_SO_4_, where the highest protein precipitation (3.0 ± 1.02 mg/ml) was observed at 80% (NH_4_)_2_SO_4_, as shown in Table 4, Fig. 4. These data were similar to the study achieved by Anu *et al*. [24], who reported that the 60% (NH_4_)_2_SO_4_ was the best concentration for cellulase activity. Many laboratories use (NH_4_)_2_SO_4_ for protein precipitation via the salt-out phenomena. Continuous protein precipitation as (NH_4_)_2_SO_4_ concentration is increased over time is evidence of the salt's efficacy in this setting. Cellulase enzyme activity is significantly increased at certain salt concentrations because the salt facilitates the formation of ion pairs and other interactions that improve the structural stability of the enzyme [46]. In the current study, when salt concentrations reached levels above 60%, cellulase activity was observed to decrease due to a phenomenon called salt inhibition or salt-induced enzyme inactivation. At high salt concentrations, the enzyme's structure and function could be disrupted, impacting the enzyme’s active sites, promoting protein aggregation, and altering the enzyme's conformation. As a result, the enzyme's catalytic activity decreased, leading to reduced efficiency [47].

Here, the specific activity of cellulase was measured after concentrating by dialysis bag against distilled water followed by sucrose, where the cellulase activity was 22.86 U/mg, as shown in Table 4. In a similar study, the specific activity of the cellulase enzyme from *Bacillus* sp. was 41.25 IU/mg after dialysis [48]. In the current study, the Sephadex G-100 was used to concentrate the purified cellulase enzyme, which was collected on 10 fractions, and the highest cellulase activity was 92.02 U/mL with a specific activity of 110.95 U/mg, as shown in Table 5.

### Cellulase Enzyme Characterization

**Molecular Weight and Amino Acid Content of Cellulase**

The *B. subtilis*-mediated biosynthesis of cellulase enzyme contains 15 amino acids as represented in Table 6, where these amino acids start with aspartic acid followed by threonine, serine, glutamic, glycine, alanine, valine, methionine, isoleucine, leucine, phenylalanine, histidine, lysine, and proline. The results showed that the highest values were 160 and 180 mg/ml for glycine and proline, respectively, while the lowest value of 10 mg/l was recorded for threonine, valine, isoleucine, leucine, and histidine respectively. The composition of amino acids in the cellulase enzyme produced by *B. subtilis* can provide valuable insights into the enzymatic properties and ecological adaptation of the bacteria to the isolation site. The high concentration of the amino acids glycine and proline in cellulase may be due to the special features of these two relatively simple amino acids. Owing to its short size and lack of a side chain, glycine can fit into the enzyme's active site with ease. Because of its adaptability, it may take part in a wide range of interactions and help stabilize the enzyme's structure, both of which improve its catalytic performance [49]. Moreover, proline is unique among amino acids due to its cyclic structure, which creates rigidity in protein structures. It is often found in turns and loops of protein chains, helping to stabilize the enzyme's three-dimensional structure and improving its thermal stability under extreme conditions [50], such as those found in the current study (hot spring samples used for bacterial isolation).

**Fig. 5:**
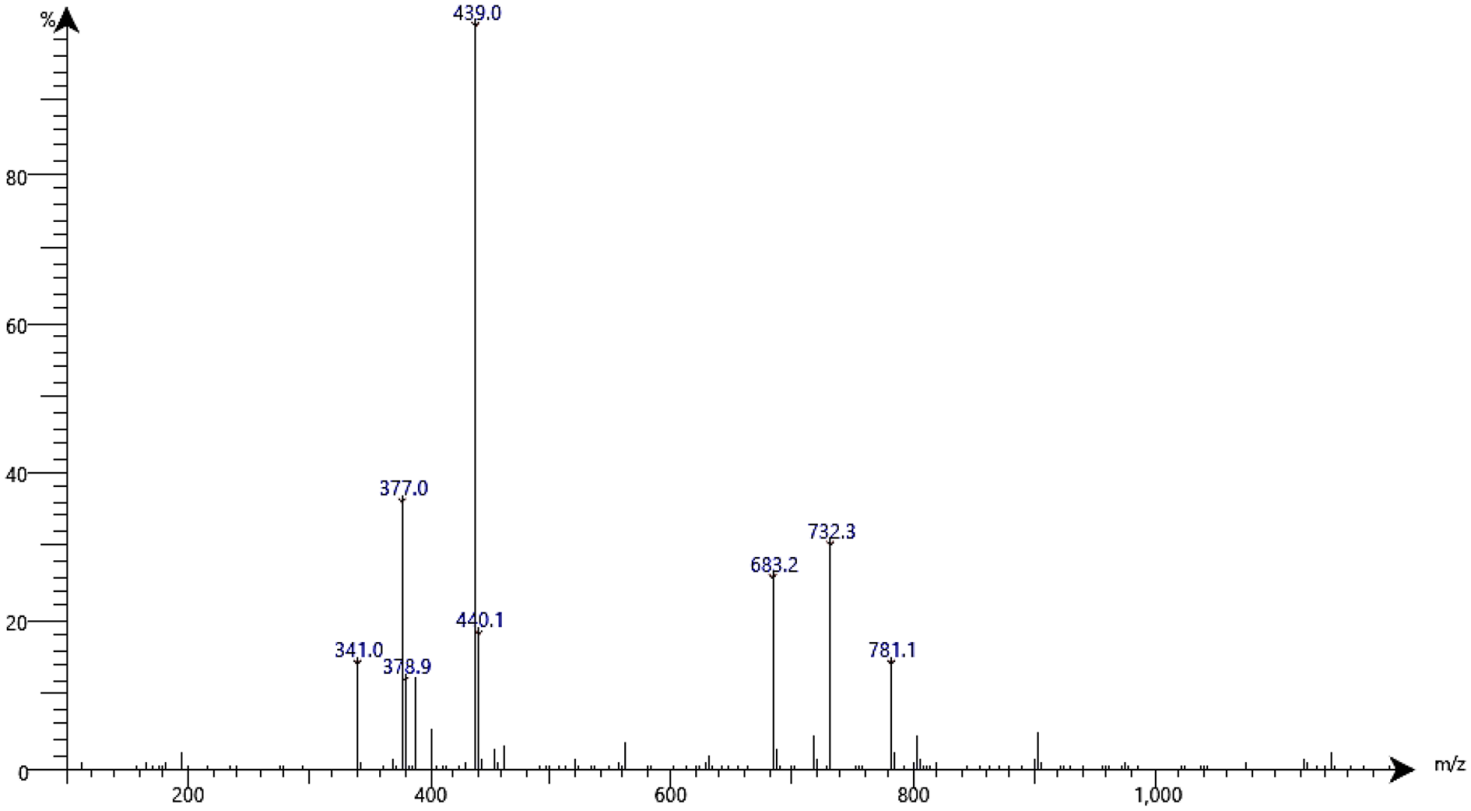
TLC analysis to detect the molecular weight of the cellulase enzyme produced by *B. subtilis* F3.

The enzyme's function and its adaptation to extreme environments may be responsible for the low concentrations of some amino acids. In the current study, the presence of larger amino acids (threonine, valine, isoleucine, leucine, and histidine) that contain bulky side chains may influence the confirmation of enzymes and hinder their stability or activity under extreme condition [51]. The high-temperature environment, which in the current study is a hot spring, can exert selective pressure on enzymes produced by microorganisms, favoring those with enhanced thermal stability and activity [52]. Glycine and prolinés properties make them well-suited for these conditions, allowing the enzyme to maintain its functionality and stability even at elevated temperatures. Overall, the high concentrations of glycine and proline in the cellulase enzyme indicate that it has adapted over time to survive in the harsh environment of hot spring waters. Hot spring conditions are characterized by heightened temperatures; hence the presence of these particular amino acids likely plays a vital role in improving the enzyme's efficiency and sustaining its functionality at these higher temperatures. In the current study, the molecular weight of the cellulase enzyme produced by *B. subtilis* strain F3 in this study was 439 KDa as shown in Fig. 5.

### Effect of pH and Temperature on Cellulase Activity

**Fig. 6:**
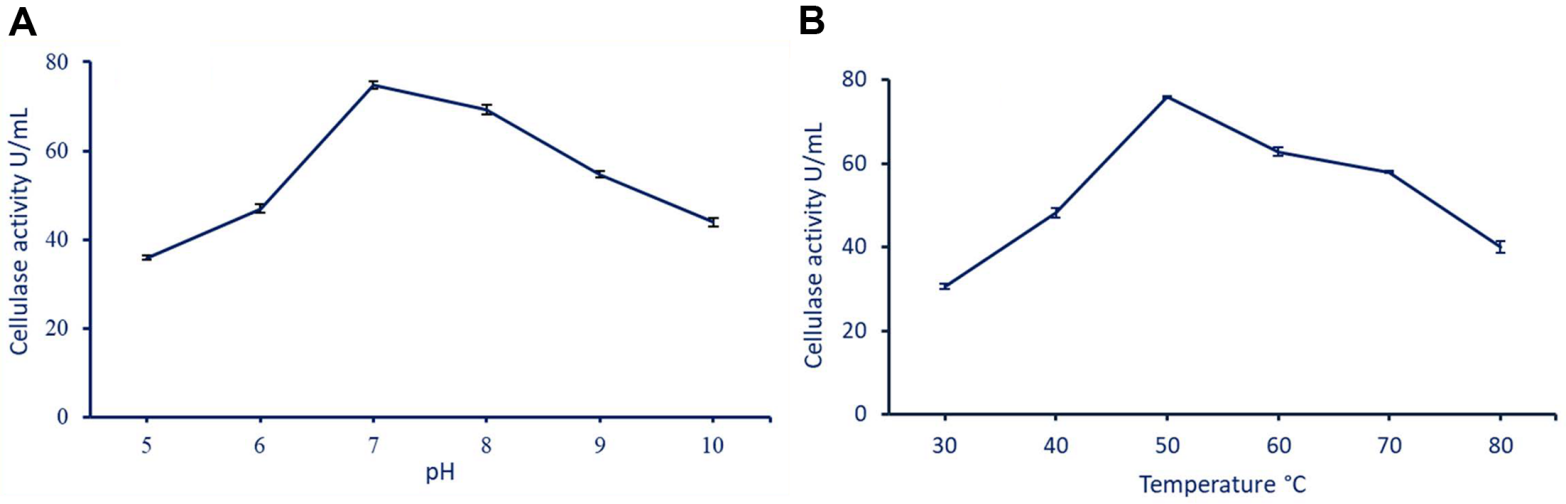
Effect of various pH values (A) and temperature degrees (B) on the activity of cellulase enzyme.

For characterization of cellulase obtained from *B. subtilis* strain F3, it was subjected to different pH values, *i.e.*, 5, 6, 7, 8, 9, and 10. According to the results from this study, we observed that the optimum pH that gives the highest cellulase activity (74.8 ± 0.8 U/ml) was 7. Interestingly, the activity decreased with increasing or decreasing the pH value as shown in Fig. 6A. This observation refers to the critical role of pH in the catalytic potential and structural integrity of cellulase enzymes. The highest activity in normal pH could be attributed to the normal pH value (6.7–7.14) at the site of the collected samples. In a similar study, the optimal pH for cellulase activity produced by *Bacillus* strains was in the range of 7–8 with an increase in their activity of more than 60% compared to other pH values [53]. Since the ideal pH for cellulase activity is enzyme-specific, it can change depending on the type and source of cellulase [54]. The ionization states of amino acid residues within the active site are strongly influenced by the pH of the surrounding environment, which in turn impacts the enzyme's interaction with its substrate and overall catalytic activity.

The purified cellulase was most active at pH 7, which is close to the neutral range, due to different factors such as the environment surrounding an enzyme's active site being extremely pH dependent. Some amino acid residues in the active site may have their ionization state optimized at pH 7, allowing for more efficient substrate binding and catalysis [55]. Also, at neutral pH, proteins are more stable, leading to the enzyme's structural integrity being best preserved and substrate interaction [56]. The concentration of hydrogen ions in a solution is affected by the pH of the solution. Hydrogen bonding and electrostatic interactions between the enzyme and substrate are disrupted at acidic or alkaline pH extremes, decreasing enzyme-substrate binding and catalysis [57].

In the present study, through measurement of the cellulase activity, *B. subtilis* F_3_ was measured after incubation at different temperatures ranging from 30°C to 80°C to detect the effect of temperature on cellulase activity. The results demonstrated that the optimum temperature for maximum cellulase activity (75.9 ± 0.3 U/ml) was 50°C, as shown in Fig. 6B. Conversely, the cellulase enzyme's stability and activity exhibit a decline when temperatures fall below or surpass this threshold. Interestingly, the temperature of the sample site was in the range of 40° to 45°C. A comparable investigation revealed that the cellulase enzyme derived from *B. licheniformis* C55 remained stable, with its peak activity realized at 50°C [53]. On the other hand, the optimum temperature for maximum stability and activity of purified cellulase obtained from *B. licheniformis* WBS1 and *Bacillus* sp. WBS3 was 60°C [58]. The thermostability of purified cellulase enzymes give it various advantages in industry, such as high desirability and competitiveness, reduced reaction times, minimized undesirable reactions, longer enzyme lifespan, and flexibility in applications [59, 60].

### Effect of Some Metal Ions on Cellulase Activity

**Fig. 7:**
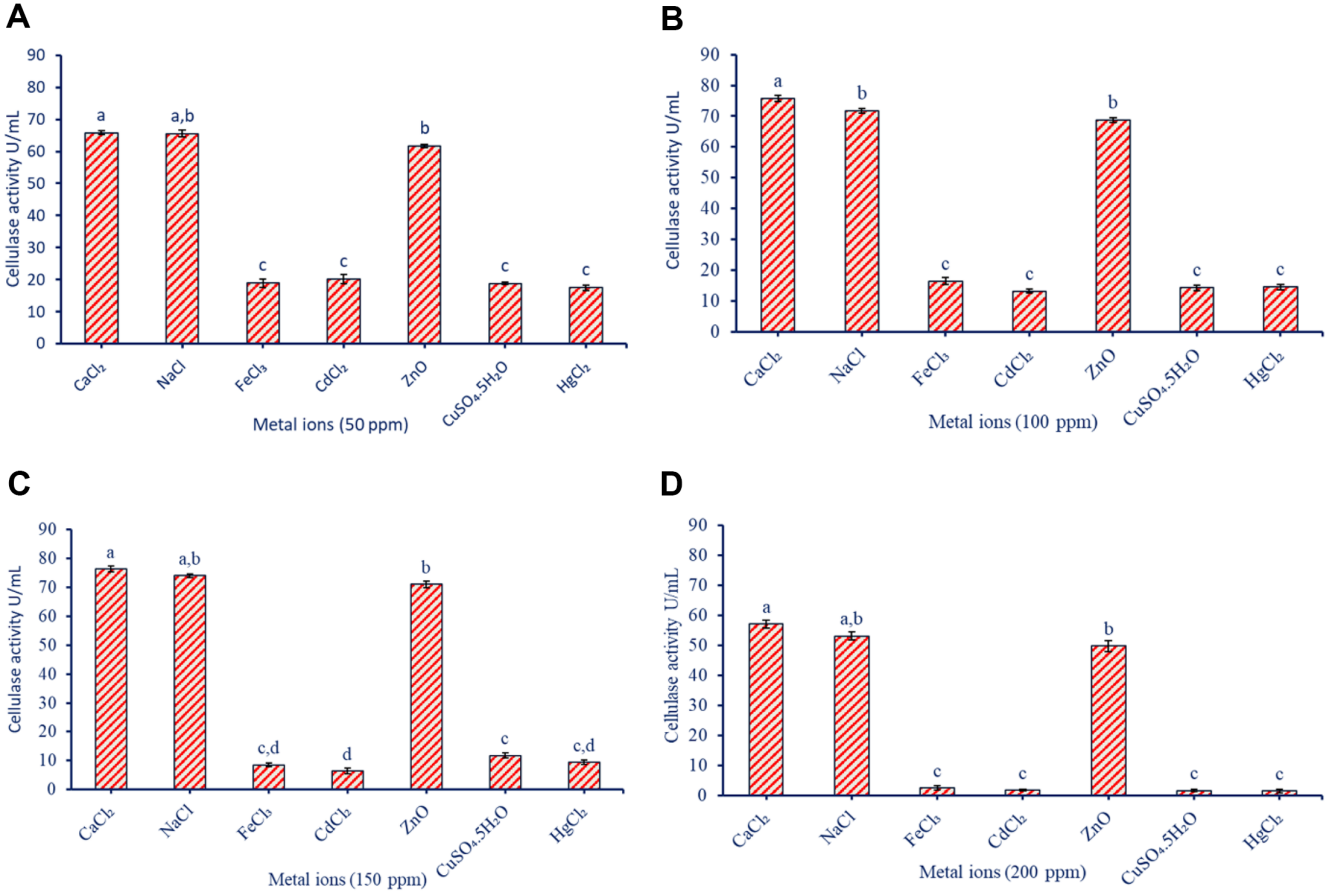
Effect of various metal ions (CaCl_2_, NaCl, ZnO, CdCl_2_, CuSO_4_·5H_2_O, FeCl_3_, and HgCl_2_) at different concentrations (50, 100, 150, and 200 ppm) on the activity of cellulase enzyme produced by *B. subtilis* F3. A to D denotes the effect of 50, 100, 150, and 200 ppm of metal ions respectively on the enzyme activity.

Metal ions play an important role in the activation or inhibition of purified cellulase enzymes. In the current study, we investigated the effect of different metal ions including CaCl_2_, NaCl, ZnO, CdCl_2_, CuSO_4_.5H_2_O, FeCl_3_, and HgCl_2_ with varied concentrations (50, 100, 150, and 200 ppm) on the activity of cellulase enzyme secreted by *B. subtilis* F3 (Fig. 7). As shown, some metal ions, such as CaCl_2_, NaCl, and ZnO, enhanced the activity of cellulase enzyme, whereas other metals, CdCl_2_, CuSO_4_.5H_2_O, FeCl_3_, and HgCl_2_, have an inhibitory effect on the cellulase activity. Data analysis showed that the activity of cellulase enzyme increased as the concentration of CaCl_2_, NaCl, and ZnO increased until a concentration of 150 ppm. At this concentration (150 ppm), the cellulase activity was 76.4 ± 0.9, 74.1 ±0.7, and 71.04 ± 1.2 U/ml, for CaCl_2_, NaCl, and ZnO, respectively. With high concentrations (200 ppm) of these metals, the cellulase activity was decreased. On the other hand, the activity of cellulase produced by bacterial strain F3 was negatively affected with varied concentrations of inhibited FeCl_3_, CdCl_2_, CuSO_4_.5H_2_O, and HgCl_2_. As shown, the activity of cellulase enzyme was highly decreased from 18.9 ± 1.4, 20.3 ± 1.4, 18.8 ± 0.4, and 17.5 ± 0.9 U/ml at a concentration of 50 ppm to 2.7 ± 0.71.8 ± 0.3, 1.7 ± 0.7, and 1.6 ± 0.6 U/ml at a concentration of 200 ppm for metal ions of FeCl_3_, CdCl_2_, CuSO_4_.5H_2_O, and HgCl_2_, respectively (Fig. 7A-7D). Ca^2+^ and Zn^2+^ ions have stabilizing effects on the enzyme structure and function, acting as co-factor, and improving the appropriate folding, which may explain why CaCl_2_ and ZnO improve the enzyme's stability and activity [61, 62]. Also, NaCl is a prevalent salt known to impart enzyme stability and establish a conducive milieu characterized by appropriate ionic strength [63]. Additional other metals (Cd^2+^, Cu^2+^, Fe^3+^, and Hg^2+^) exert an adverse impact on the activity of cellulase. This occurs as a consequence of these metals disrupting the enzyme's structural integrity, perturbing crucial interactions, generating reactive oxygen species (ROS) leading to oxidative harm, and subsequently resulting in enzyme deactivation [64]. In a similar study, the activity of purified cellulase enzyme secreted by bacteria strain *B. sphaericus* CE-3 was enhanced in the presence of Mn^2+^ ion, whereas it was inhibited by other metals including Ba^2+^, Co^2+^, Hg^2+^, Pb^2+^, Cu^2+^, Sr^2+^, and Fe^2+^ [65].

### Biopolishing of Cotton Fabric Using Cellulase Enzyme

The obtained data revealed that the weight loss percentages of the cotton fabric were proportional to the concentration of cellulase enzyme and the time of treatment (Table 7). As shown, the weight loss percentages of fabric were 2.04 ± 0.2, 1.7 ± 0.1, and 2.7 ± 0.1% due to treatment with cellulase enzyme at concentrations of 0.25, 0.5, and 1.0% for 30 min. Moreover, the weight loss percentages increased with the time of treatment. For instance, the weight loss of cotton fabrics increased to 3.4 ± 0.2, 4.2 ± 0.1, and 3.6 ± 0.8% after 90 min of treatment with concentrations of 0.25, 0.5, and 1.0%, respectively (Table 7). Weight loss by cotton fabrics is an important result and evidence of good biopolishing, or the process of removing fibrils or protruding fibers, from the surface of cotton fabric. The presence of these fibrils produces pilling, which in turn diminishes the smoothness of the fabric. Enzymes or chemicals degrade these fibrils, reducing the fabric's overall weight when the surplus is eliminated [14]. The application of cellulase enzyme to cotton fabrics possesses the capability to permeate the fabric's structure, resulting in the degradation of fibers located between the yarn and the inner layers of the fabric. This ultimately culminates in a reduction of the fabric's weight [16].

To polish cotton fabrics, various chemical substances such as acetic acid (CH_3_COOH), hydrogen peroxide (H_2_O_2_), ethylenediaminetetraacetic acid (EDTA), sodium hypochlorite (NaCIO), and surfactant have been utilized. These compounds have drawbacks that limit their applicability, such as the fact that high concentrations of hydrogen peroxide (H_2_O_2_) or continuous exposure to it can cause damage to fabrics, thereby lowering both their strength and durability [66]. Besides the negative impact of H_2_O_2_ and NaCIO on humans and the environment, it can cause color fading in cotton fabrics [67]. NaCIO has the potential to induce irritation of the skin and eyes, along with the capacity to harm gastrointestinal and respiratory tissues [68]. Similarly, EDTA is employed to chelate metal ions, forming complexes that could negatively affect the ecosystem if not properly treated or disposed of [69]. The discernible odor resulting from the utilization of CH_3_COOH might prove undesirable during the polishing procedure. Moreover, increasing amounts of pollution are produced by alkaline bleaching processes, whether they are traditional or have been modified over time. In contrast, pre-treating cotton fabric with enzymes before bleaching is a successful alternative method that can be used instead of bleaching. This alternative process achieves approximately equal results in terms of the brightness and color intensity of the cloth while having much fewer negative effects on the environment [70]. As a result, researchers are primarily focused on employing substances that are both safe and environmentally friendly for the bleaching of cotton fabrics. Cellulase enzymes sourced from various biological entities, such as bacteria, actinomycetes, and fungi, offer a viable alternative for this purpose. In a similar study, the weight of cotton fabrics was reduced as the enzyme concentration and treatment times were heightened. However, this weight loss was not nearly as significant as the weight decrease that was accomplished with the use of chemical substances such as H_2_O_2_ [71].

## Conclusion

In this study, the optimal conditions for maximum cellulase production using *B. subtilis* F3 strain were determined as follows: 24 h incubation, pH 8, temperature of 45°C, inoculum size of 1%, with 5 g/l of peptone as the nitrogen source, and 7.5 g/l of CMC powder. Statistical analysis using Tukey HSD at *p* < 0.05 verified these conditions. Additionally, a Box-Behnken design was employed to pinpoint the optimal conditions for large-scale cellulase production, yielding the highest activity of 63.11 U/ml at a temperature of 43°C, pH 8, peptone concentration of 5 g/l, inoculum size of 1.5%, CMC concentration of 6.5 g/l, and a 24 h incubation period. The cellulase enzyme's molecular weight was determined to be 439 KDa, with amino acid analysis revealing the presence of 15 amino acids in varying quantities. Interestingly, the cellulase enzyme showed promise as a biopolishing agent for cotton fabric. These findings hold significant implications for industrial applications, particularly in environmentally friendly biopolishing processes that reduce the reliance on harmful chemicals. Overall, this investigation provides valuable insights for future fermenter scale-up experiments and advancements in cellulase enzyme manufacturing.

## Figures and Tables

**Table 1:** Six factors and their three levels are used in BBD for cellulase production.

Factor name		Units	-1	0	+1
Temperature	A	°C	43	45	47
pH	B	-	7.5	8	8.5
Inoculum size	C	%	0.5	1	1.5
Peptone	D	g/l	3	5	7
Incubation period	E	h.	20	24	28
CMC	F	g/l	6.5	7.5	8.5

**Table 2:** Qualitative screening test for production of extracellular cellulase enzyme at 45°C for 24 h.

Bacterial isolate	Cellulase	Bacterial isolate	Cellulase	Bacterial isolate	Cellulase	Bacterial isolate	Cellulase
C1	-	C9	+	E1	+	F2	++
C2	-	C10	++	E2	++	F3	+++
C3	++	D1	-	E3	-	F4	+
C4	-	D2	-	E4	+	F5	+
C5	-	D3	-	E5	-	F6	-
C6	++	D4	-	E6	+	F7	++
C7	-	D5	++	E7	-	F8	++
C8	-	D6	++	E8	++	F9	-
C9	-	D7	++	E9	++	F10	-
C10	-	D8	+	F1	++	F11	++

(-) = negative, (+) = 10–20 mm, (++) = 20–30 mm, (+++) = 30–40 mm

**Table 3:** Various runs for production of cellulase enzyme using *B. subtilis* F3 after employing Box-Behnken design.

Run	Temp.	pH	Peptone	Inoculum Size	CMC Conc	Incubation period (h)	Enzyme activity (U/ml)	Predicted
**1**	47	8	5	0.5	6.5	24	46.75	46.9544
**2**	47	8	7	1	7.5	28	38.42	35.1848
**3**	47	8	7	1	7.5	20	40.44	40.0777
**4**	43	8.5	5	0.5	7.5	24	49.49	45.6610
**5**	47	8.5	5	1.5	7.5	24	42.73	39.4515
**6**	45	8.5	5	1	8.5	28	35.49	55.1729
**7**	45	8	3	1.5	7.5	20	48.47	39.3944
**8**	47	8	3	1	7.5	28	37.19	41.6923
**9**	45	8	7	1.5	7.5	28	42.81	51.9187
**10**	43	8	3	1	7.5	20	41.68	57.5133
**11**	45	8	3	0.5	7.5	28	39.74	44.2854
**12**	43	8	5	0.5	6.5	24	58.39	51.2662
**13**	47	8	3	1	7.5	20	39.44	44.2979
**14**	45	8	3	1.5	7.5	28	40.29	45.1762
**15**	45	7.5	7	1	8.5	24	52.88	40.6715
**16**	45	8	5	1	7.5	24	59.37	49.0996
**17**	45	8.5	5	1	6.5	28	44.43	55.9346
**18**	45	8.5	3	1	6.5	24	49.84	45.8021
**19**	47	7.5	5	0.5	7.5	24	45.42	52.3104
**20**	45	8.5	5	1	6.5	20	47.77	52.2837
**21**	43	8	3	1	7.5	28	40.29	57.5133
**22**	45	7.5	5	1	8.5	20	49.42	56.8946
**23**	45	7.5	3	1	6.5	24	58.45	57.5133
**24**	47	8	5	1.5	8.5	24	42.86	46.8696
**25**	45	8.5	7	1	8.5	24	50.26	57.5133
**26**	43	8	5	1.5	8.5	24	52.26	41.9419
**27**	45	8	5	1	7.5	24	59.47	38.8488
**28**	45	7.5	3	1	8.5	24	60.24	45.5421
**29**	45	8	5	1	7.5	24	59.37	59.8637
**30**	45	7.5	5	1	6.5	20	47.18	48.7913
**31**	45	8	5	1	7.5	24	59.37	49.1229
**32**	45	8	7	0.5	7.5	28	40.16	57.5133
**33**	45	7.5	5	1	8.5	28	36.72	42.5594
**34**	47	8	5	1.5	6.5	24	42.18	45.0638
**35**	43	8	5	1.5	6.5	24	63.11	49.4915
**36**	47	8	5	0.5	8.5	24	45.27	47.2823
**37**	43	8	5	0.5	8.5	24	52.76	43.3885
**38**	45	8	5	1	7.5	24	59.37	59.0604
**39**	43	8	7	1	7.5	28	42.78	45.8946
**40**	47	7.5	5	1.5	7.5	24	44.70	50.3371
**41**	43	8	7	1	7.5	20	47.22	56.2104
**42**	45	8	7	1.5	7.5	20	47.26	41.3387
**43**	45	8	3	0.5	7.5	20	40.39	41.8762
**44**	45	8.5	7	1	6.5	24	55.25	47.8215
**45**	47	8.5	5	0.5	7.5	24	46.75	49.2946
**46**	43	8.5	5	1.5	7.5	24	49.49	53.1387
**47**	43	7.5	5	1.5	7.5	24	55.63	57.5133
**48**	45	7.5	5	1	6.5	28	38.17	46.6962
**49**	45	8.5	5	1	8.5	20	44.58	46.9544
**50**	45	8	7	0.5	7.5	20	49.49	35.1848
**51**	43	7.5	5	0.5	7.5	24	46.97	40.0777
**52**	45	7.5	7	1	6.5	24	53.13	45.6610
**53**	45	8	5	1	7.5	24	48.13	39.4515
**54**	45	8.5	3	1	8.5	24	47.17	55.1729

**Table 4:** Partial purification of cellulase enzyme synthesized by *B. subtilis* strain F3.

Sample	Total volume (ml)	Total protein (mg)	Specific activity (U/mg)	Purification fold	Yield %
Crude enzyme	50	219.45	10.05	1	100
(NH_4_)_2_SO_4_ 60%	35	122.01	15.95	1.59	92.24
Dialysis process	22	63.31	22.86	2.27	65.62
Sephadex G-100	50	27.83	54.20	5.39	68.41

**Table 5:** Fractionation pattern of cellulase enzyme synthesized by *B. subtilis* strain F3 using Sephadex G100 column chromatography technique.

Fraction No.	Cellulase activity (U/ml)	Protein content (mg/ml)	Specific enzyme activity (U/mg)
**1**	0	0	0
**2**	1.76	12.99	0.135489
**3**	16.23	9.11	1.781559
**4**	42.12	1.98	21.27273
**5**	80.45	1.23	65.4065
**6**	92.09	0.83	110.9518
**7**	40.71	0.64	63.60938
**8**	20.34	0.49	41.5102
**9**	6.10	0.31	19.67742
**10**	1.88	0.25	7.52

**Table 6:** Amino acids and their concentration (mg/ml) present in cellulase enzyme secreted by *B. subtilis* F3.

Amino acid	Amount (mg/ml)	Amino acid	Amount (mg/ml)
Aspartic	20	Methionine	20
Threonine	10	Isoleucine	10
Serine	20	Leucine	10
Glutamic	30	Phenylalanine	30
Proline	180	Histidine	10
Glycine	160	Lysine	20
Alanine	60	Arginine	20
Valine	10		

**Table 7:** Percentage reduction in weight of cotton fabrics treated with cellulase enzyme derived from *Bacillus subtilis* F3.

Run number	Cellulase concentration (%)	Time (min)	Weight loss percentage of cotton fabric after cellulase treatment (%)
1	0.25	30	2.04±0.2
2	0.25	60	2.4±0.4
3	0.25	90	3.4±0.2
4	0.5	30	1.7±0.1
5	0.5	60	3.3±0.3
6	0.5	90	4.2±0.1
7	1	30	2.7±0.1
8	1	60	3.5±0.2
9	1	90	3.6±0.8
